# Cuproptosis scoring model predicts overall survival and assists in immunotherapeutic decision making in pancreatic carcinoma

**DOI:** 10.3389/fgene.2022.938488

**Published:** 2022-08-31

**Authors:** Tijun Liu, Qing Liu, Yongju Wang, Rong Yang, Fang Tian

**Affiliations:** ^1^ Department of Rehabilitation Medicine, Xiantao First People’s Hospital Affiliated to Yangtze University, Xiantao, China; ^2^ Department of Oncology, Xiantao First People’s Hospital Affiliated to Yangtze University, Xiantao, China

**Keywords:** pancreatic carcinoma, cuproptosis, overall survival, immunotherapy, risk score

## Abstract

**Background:** Cuproptosis is a newly identified form of non-apoptotic cell death that is associated with the progression and treatment responses in pancreatic adenocarcinoma (PAAD). However, its impact on oncology and tumor microenvironment (TME) remains unclear.

**Methods:** Hub genes were identified using least absolute shrinkage and selection operator (LASSO) Cox regression for 25 newly reported cuproptosis-related regulators and subjected to stepwise regression to obtain cuproptosis-related score (CuRS). Additionally, the clinical significance, functional status, role on TME, and genomic variation of CuRS were further examined systematically.

**Results:** A CuRS model incorporating TRAF2, TRADD, USP21, FAS, MLKL, TNFRSF10B, MAPK8, TRAF5, and RIPK3 was developed. The stability and accuracy of this risk model as an independent prognostic factor for PAAD were confirmed in the training and external validation cohorts. Patients in the high-CuRS group had “cold” tumors with active tumor proliferation and immunosuppression, whereas those in the low-CuRS group comprised “hot” tumors with active immune function and cell killing capacity. Additionally, patients in the high-CuRS group carried fewer genomic copy number variations (CNVs) and greater somatic mutations. Furthermore, patients in the low- and high-CuRS groups exhibited increased sensitivity to immunotherapy and chemotherapy, respectively.

**Conclusion:** We developed and validated a robust CuRS model based on cuproptosis to assess patients’ prognoses and guide clinical decision-making. Overall, the findings of this study are expected to contribute to the comprehensive understanding of cuproptosis and facilitate precise treatment of PAAD.

## Introduction

Pancreatic adenocarcinoma (PAAD) is a malignancy with one of the poorest prognoses, thus leading to extremely high mortality rates. Although the incidence of pancreatic cancer is low, it is the fourth leading cause of cancer-related deaths (Siegel et al., 2021). One reason for unfavorable prognoses in patients with PAAD is the insensitivity to most therapies, including chemotherapy, radiotherapy, and immunotherapy (Schneider et al., 2005). Therefore, currently, surgical resection is the only feasible option. In most malignancies that respond to treatment, responses to chemotherapy and radiotherapy are realized through apoptosis induction in cancer cells (Schulze-Bergkamen and Krammer, 2004). Apoptosis evasion is a hallmark of all cancers and a plethora of molecular mechanisms have evolved to resist apoptosis, especially in PAAD (Hamacher et al., 2008). Despite years of extensive research worldwide, the prognoses of patients with PAAD remain unfavorable. Therefore, it is crucial to identify new underlying mechanisms to improve patients’ prognoses and develop new therapies.

Stressors, including DNA damage, protein misfolding, or cytoskeleton disruption, can lead to cell death mediated by the inactivation of apoptosis-related pathways (Maiuri et al., 2007). Iron catalyzes the formation of toxic membrane lipid peroxides to mediate a unique form of non-apoptotic cell death-ferroptosis, as evidenced by recent findings (Kagan et al., 2017). Additionally, copper overload can lead to a novel cell death mechanism, namely, cuproptosis (Tsvetkov et al., 2022), mediated by protein acylation wherein copper directly binds to the lipidated components of the tricarboxylic acid (TCA) cycle, leading to lipid-acylated protein aggregation and loss of iron-sulfur cluster proteins, ultimately resulting in proteotoxic stress and cell death (Tsvetkov et al., 2022). These findings suggest that copper ion carriers may serve as viable therapeutic targets in cancer cells with a high respiratory rate, abundantly expressing acylated mitochondrial proteins. This new approach to killing cancer cells may be particularly effective for tumors that are naturally resistant to apoptosis (Kahlson and Dixon, 2022; Tsvetkov et al., 2022). Thus, an in-depth evaluation of cuproptosis can provide novel treatment options for PAAD.

In this study, a robust cuproptosis-related score (CuRS) model was developed and validated. This model exhibited stability and accuracy in both the training and external validation cohorts and can be used as an independent prognostic factor for PAAD. Patients in the high-CuRS group had “cold” tumors with active tumor proliferation and immunosuppression, whereas those in the low-CuRS group exhibited “hot” tumors with active immune function and cell killing capacity. Additionally, patients in the high-CuRS group carried fewer genomic copy number variations (CNVs) and greater somatic mutations. Furthermore, patients in the low- and high-CuRS groups showed increased sensitivity to immunotherapy and chemotherapy, respectively.

## Methods

### Data acquisition and pre-processing

Data from transcriptomic RNA sequencing (RNA-seq), HumanMethylation450 array, Mutect2 mutation, CNVs, and the corresponding patients’ clinical follow-up in The Cancer Genome Atlas (TCGA)–PAAD cohort were acquired from TCGA (https://cancergenome.nih.gov/). A total of 176 PAAD specimens were included after excluding patients with incomplete clinical information. Paired normal PAAD specimens and RNA-seq data from the International Cancer Genome Consortium (ICGC)–PAAD cohort (comprising 165 PAAD samples with complete clinical information) were collected from the Genotype-Tissue Expression (GTEx) database (https://xenabrowser.net/datapages/) and ICGC (https://daco.icgc.org/). Additionally, dataset E-MTAB-6134 containing 288 PAAD specimens with complete clinical information was collected from the Array Express database (https://www.ebi.ac.uk/arrayexpress).

Raw fragments per kilobase million (FPKM) data from TCGA–PAAD and ICGC–PAAD cohorts (RNA-seq data) were converted to transcripts per million (TPM) format for normalization. In addition, the microarray data were normalized using the R package, “limma”. TCGA–PAAD was used as the training cohort, whereas ICGC–PAAD and E-MTAB-6134 were used as the external validation cohorts. Subsequently, the immunotherapy cohort, IMvigor210, comprising 298 patients with uroepithelial cancer who underwent treatment with PD-L1 immunotherapy (Mariathasan et al., 2018) was obtained (http://research-pub.gene.com/IMvigor210CoreBiologies) and data were subjected to log2 normalization to assess patients’ responses to immunotherapy.

### Construction and validation of the cuproptosis-related score model

Twenty-five cuproptosis-related genes (CRGs) were collected from Tsvetkov et al. (Supplementary Table S1). Additionally, prognosis-related necrosis genes were screened by univariate COX regression analysis. To avoid omission, only genes with *p* < 0.2 were collected for further analysis. Subsequently, a LASSO penalized Cox proportional risk model was used to identify the optimal prognostic model, followed by five-fold cross-validation to assess the model’s stability. Finally, CuRS was calculated using the equation below:

$$CuRS=\sum iCoefficient\left(mRNA_{i}\right)\times Expression\left(mRNA_{i}\right)$$

The consistency index (C-index) was calculated using the R package, “survcomp”, to assess the predictive power of CuRS, where a larger C-index indicated a higher predictive accuracy of the model (Schröder et al., 2011). Additionally, patients were classified into high- and low-CuRS groups according to the median CuRS. Finally, the prognostic value of the CuRS model in the three PAAD cohorts was systematically assessed by Kaplan–Meier (KM) survival curves, univariate and multivariate Cox regression, and time-dependent ROC (tROC) curve analyses.

### Functional enrichment and immune infiltration analyses

Single-sample gene set enrichment analysis (ssGSEA) was performed to assess the activities of biological pathways enriched in the samples, including molecular markers for angiogenesis, epithelial-mesenchymal transition (EMT), myeloid inflammation, and other immune-related pathways, based on previously published molecular markers using the R package, “gsva” (Ayers et al., 2017; Gibbons and Creighton, 2018; McDermott et al., 2018; Liang et al., 2020). Hypoxia-related molecular markers were collected from Msigdb (Liberzon et al., 2011) and detailed in Supplementary Table S2. Additionally, GSEA was performed to assess the differences in KEGG pathway enrichment and treatment outcomes between the high- and low-CuRS groups; significant pathways with the criterion of *p* < 0.05 were obtained.

The infiltration abundance of 22 immune cells in the tumor samples was estimated using the R package, “CIBERSORT” (Newman et al., 2015). The immunoreactivity and tumor purity of the samples were assessed using the Estimate algorithm (Yoshihara et al., 2013). Furthermore, differences in activities among six classical immune checkpoints (CTLA-4, LAG-3, PD-1, PD-L1, PD-L2, and TIM3) were compared.

Finally, homologous recombination defect (HRD) scores, proliferation, lymphocyte infiltration signature scores, TGF-β response, indel neoantigens, and SNV neoantigens were obtained from (Thorsson et al., 2018). The immunophenoscores (IPS) of individual samples were calculated based on a previous study; high IPS indicated a stronger immune activity (Charoentong et al., 2017).

### Genomic variation landscape between the two subgroups

Mutation data were processed using the R package, “maftools”, to compare the differences in mutation burden between the high- and low-CuRS groups. The tumor mutation burden (TMB) was calculated for each patient and the high-frequency mutant genes with mutation number >5 were identified. Subsequently, the frequency differences in high-frequency mutant genes between the high- and low-CuRS groups were compared by a chi-square test and visualized using maftools (Mayakonda et al., 2018). Additionally, CNV data were preprocessed using Gistic 2.0 on the Genepattern website to identify the significantly amplified and deleted chromosomal segments and assess CNV differences on the chromosomal arms. Finally, the results for CNV events were visualized using the R package, “ggplot2.”

### Clinical value of the cuproptosis-related score model

Four first-line drugs for PAAD (5-FU, cisplatin, gemcitabine, and paclitaxel) were selected to predict the relevant half-maximal inhibitory concentration (IC_50_) for patients using the ridge regression function in the pRRophetic package. Next, the predictive accuracy of the model was assessed by ten-fold cross-validation (Geeleher et al., 2014), wherein low IC_50_ indicated high treatment sensitivity. Additionally, differentially expressed genes (DEGs) between the high- and low-CuRS groups were considered potential therapeutic targets. Subsequently, the top 300 DEGs were imported into the CMap database (https://clue.io/) to determine the potential small molecule compounds targeting these genes and elucidate their mechanisms of action. Patient responses to immunotherapy were predicted using the tumor immune dysfunction and exclusion (TIDE) algorithm (http://tide.dfci.harvard.edu) (Jiang et al., 2018). Further, the unsupervised subclass mapping algorithm (https://cloud.genepattern.org/gp/) was used to assess patient responses to anti-PD1 and anti-CTLA-4 immunotherapies based on transcriptomic expressions. Finally, the predictive performance of the CuRS model was validated in the immunotherapy cohort, Imvigor210.

### Bioinformatic and statistical analyses

All statistical analyses and plotting were performed using R software (version: 4.05). Comparisons between two groups were conducted using the Wilcoxon test and differences in proportions were compared using the chi-square test. KM survival curves and time-dependent tROC curves were plotted using the R packages, “ggsurvival” and “survivalROC”, respectively. Univariate and multivariate Cox regression analyses were performed using the R package, ‘survival’. Additionally, the R package, “rms”, was used to plot the nomogram and calibration curves, while the decision curve analysis (DCA) was performed using the “DCA” package (Vickers et al., 2008). Two-tailed *p* < 0.05 was considered statistically significant unless stated otherwise.

## Results

### Genomic landscape of cuproptosis-related genes in pancreatic adenocarcinoma

**FIGURE 1:**
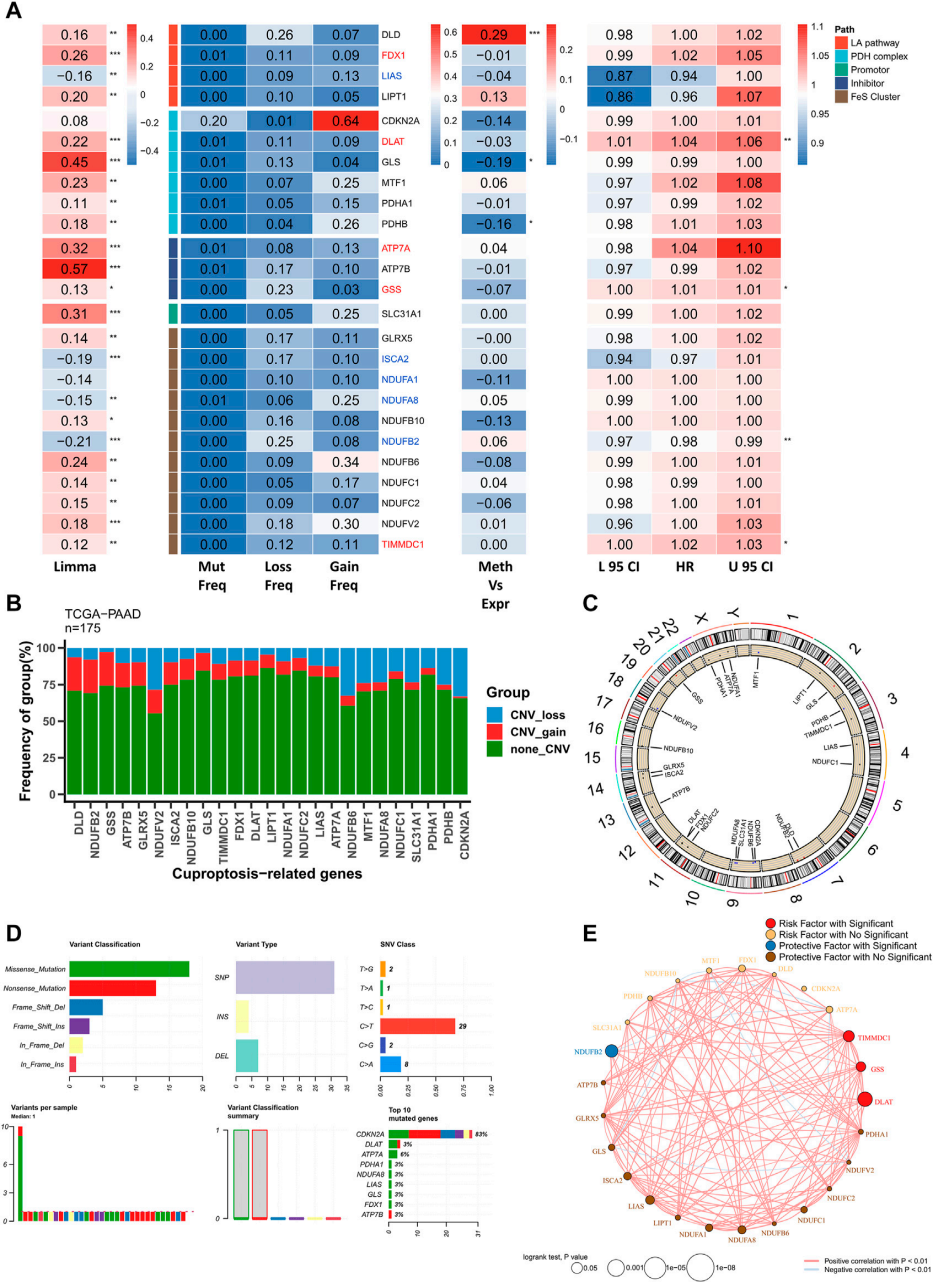
Genomic profile of CRGs in PAAD. **(A)** Heat map showing genomic variations and hazard ratios of CRGs in TCGA–PAAD; from left to right: mutation and CNV frequencies of CRGs, the correlation between DNA methylation modifications of CRGs and CRG expression; univariate Cox regression analysis showing risk ratios of FRGs. *p < 0.05, **p < 0.01, ***p < 0.001; **(B)** Bar chart showing CNV events in CRGs in TCGA–PAAD; **(C)** Circle plot showing CNV events of CRGs on chromosomes; **(D)** Summary of CRG mutation events in TCGA–PAAD; **(E)** Correlation network of CRGs (*p* < 0.01).

The multi-omics profile of CRGs in the TCGA–PAAD cohort is shown in Figure 1A. Most CRGs were upregulated in patients with PAAD and the mutation and CNV frequencies of CRGs were low. However, CDKN2A was substantially active with higher mutation and CNV frequencies. Additionally, only GLS and PHDB were significantly negatively correlated with methylation levels, suggesting that CRGs were relatively silent in PAAD and rarely regulated by other factors. Most cuproptosis-related biological processes were involved in PAAD progression. Five genes, including FDX1, DLAT, ATP7A, GSS, and TIMMDC1 were the significant risk factors for PAAD and their levels of expression were elevated in these patients. In contrast, five significant protective factors, including LIAS, ISCA2, NDUFA1, NDUFA8, and NDUFB2 were markedly low in patients with PAAD. The mutation and CNV profiles of CRGs on the chromosomes are displayed in Figures 1B,C, respectively. Moreover, a comprehensive mutation profile of CRGs is shown in Figure 1D. All CRGs exhibited lower mutation frequencies except for DKN2A. In addition, the most prevalent mutation type was the nonsense mutation. Finally, the correlation network of CRGs was constructed. As most CRGs were significantly positively correlated with each other, only the pairs with *p* < 0.01 are shown (Figure 1E).

### Construction of a cuproptosis-related gene-based risk model

**FIGURE 2:**
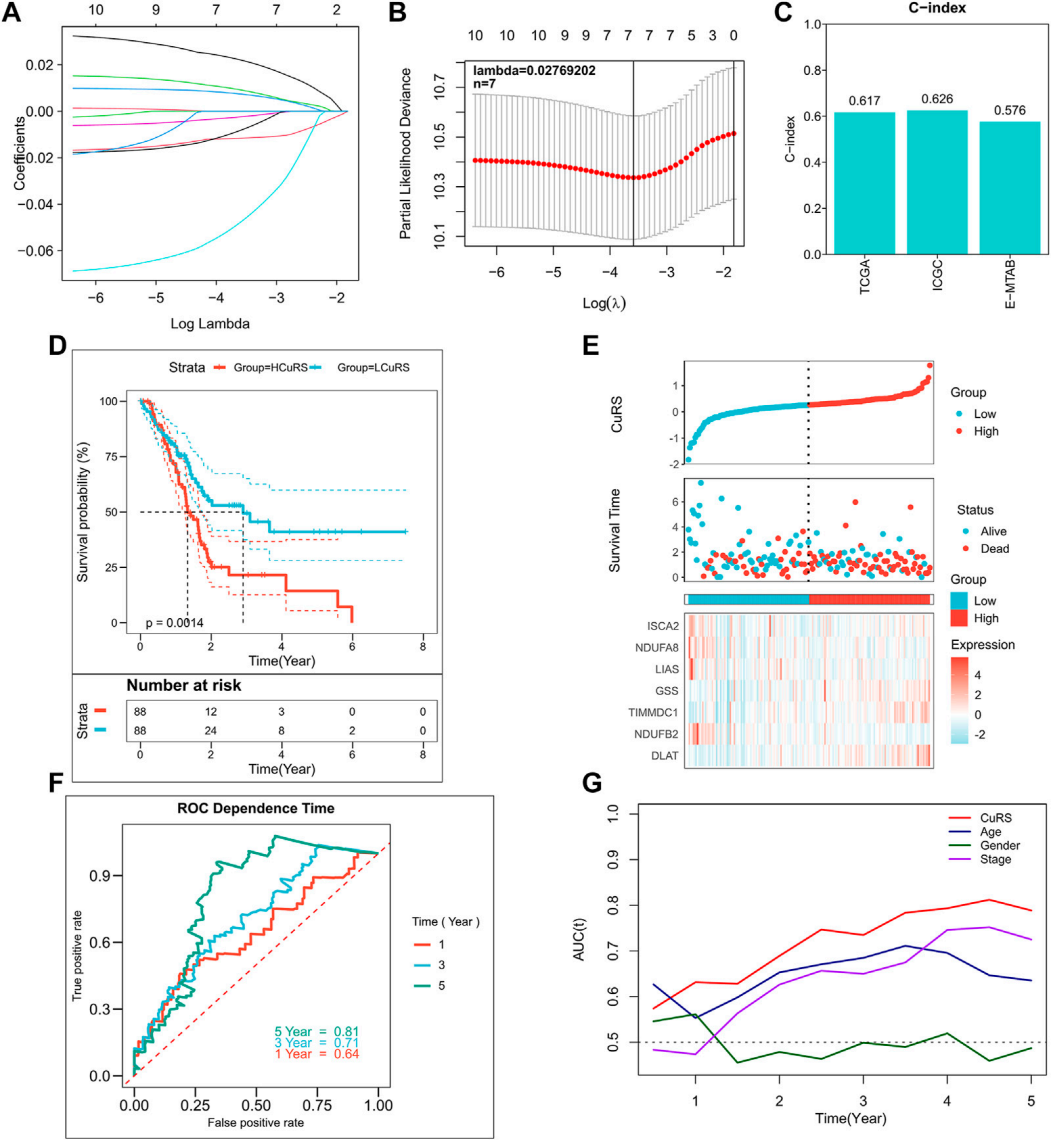
Construction of the CRG-based risk model. **(A)** Construction of the LASSO model; **(B)** Construction of the optimized model incorporating 7 CRGs based on the optimal lambda; **(C)** C-index of the optimized model in TCGA, ICGC, and E-MTAB cohorts; **(D)** KM survival curves for high- and low-CuRS groups in TCGA cohort; **(E)** Survival status of patients in TCGA cohort and expression of the model genes; **(F)** 1-, 3-, 5-, and 8-year ROC curves for CuRS in TCGA cohort; **(G)** CuRS model and tROC curves of clinical characteristics in TCGA cohort.

A total of 10 CRGs, including ATP7A, DLAT, FDX1, GSS, LIAS, ISCA2, NDUFA1, NDUFA8, NDUFB2, and TIMMDC1, were screened as candidate genes for the model based on the threshold of *p* < 0.2. The Cox regression results are detailed in Supplementary Table S3. The optimal combination of prognostic factors was screened using the LASSO model, and the optimized model comprising seven prognostic CRGs was obtained according to Lambda = 0.02769202 (Figures 2A,B). This model showed good accuracy in both the training and the external validation cohorts (TCGA: 0.617; ICGC: 0.626; GEO: 0.576) (Figure 2C). Additionally, the CuRS model was constructed according to the equation, $Risk\;Score=\sum iCoefficient\left(mRNA_{i}\right)\times Expression\left(mRNA_{i}\right)$ , and the LASSO coefficients for the model genes are listed in Supplementary Table S4. Results of the survival analysis suggested that patients in the high-CuRS group showed significantly lower survival rates than those in the low-CuRS group (Figure 2D; *p* = 0.0014). Figure 2E illustrates the distribution of CuRS and model genes in TCGA cohort. The 1-, 3-, and 5-year AUCs of the model were 0.64, 0.71, and 0.81, respectively (Figure 2F). In addition, the results of the tROC analysis suggested that CuRS was the best predictor (Figure 2G). Subsequently, the predictive performance of the model was verified in a validation cohort. Survival analysis suggested significantly poorer survival among patients in the high-CuRS group (Supplementary Figures S1A,B, *p* < 0.01). The distribution of CuRS and model genes in the ICGC and GEO cohorts are shown in Supplementary Figures S1C,D. Overall, the CuRS model showed satisfactory predictive power in both the external validation cohorts [ICGC: 1-, 3-, and 5-year AUCs of 0.64, 0.68, 0.70, respectively (Supplementary Figure S1E); GEO: 1-, 3-, and 5-year AUCs of 0.64, 0.59, 0.56, respectively (Supplementary Figure S1F)].

### Predictive independence of the cuproptosis-related score model

**FIGURE 3:**
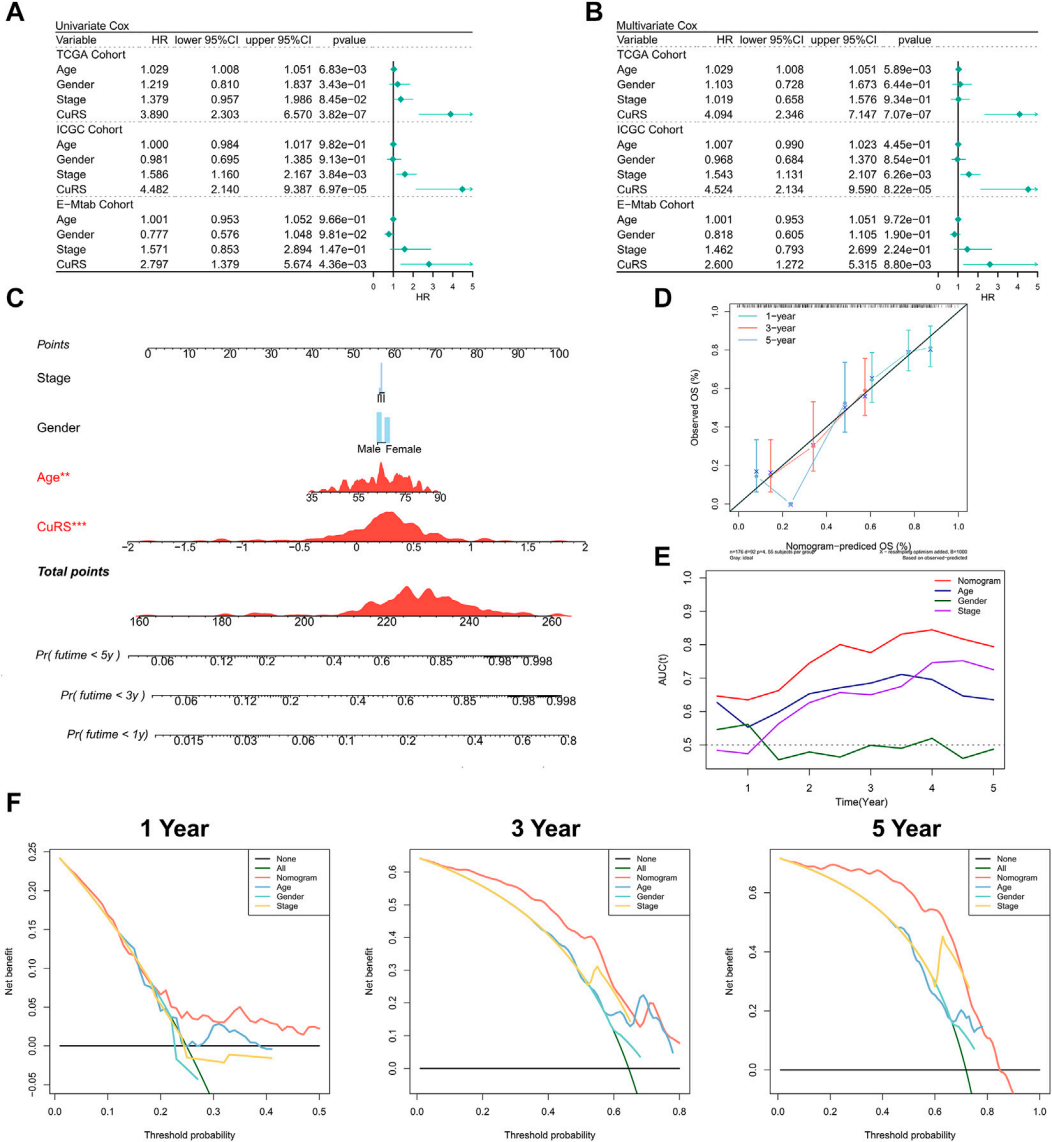
Validation of the CuRS model. **(A)** Univariate Cox regression analysis of OS in TCGA, ICGC, and E-MTAB datasets; **(B)** multivariate Cox regression analysis of OS in TCGA, ICGC, and GE datasets; **(C)** FRS-based nomogram; **(D)** Calibration curves for the nomogram; **(E)** Nomogram and tROC curves for clinical characteristics; **(F)** 1-, 3-, and 5-year DCA curves for the nomogram.

The relationship of CuRS with clinical characteristics and prognoses of patients was analyzed using univariate and multivariate Cox regression analyses. Results of the univariate Cox regression analysis suggested that CuRS could serve as an independent prognostic indicator in both the training and validation cohorts (*p* < 0.01) (Figure 3A). Additionally, multivariate Cox regression analysis showed that CuRS remained an independent prognostic factor for OS in both the training and validation cohorts after correction for other clinical characteristics (*p* < 0.01) (Figure 3B). Furthermore, subgroup analysis suggested that CuRS remained a reliable prognostic factor in different clinical subgroups (Supplementary Figure S2). Subsequently, the nomogram was constructed to better quantify the risk of patients with PAAD (Figure 3C). The correction curve of the nomogram showed excellent 1-, 3-, and 5-year stability and accuracy (Figure 3D). In addition, tROC analysis confirmed that the nomogram was the best predictor relative to all other clinical characteristics (Figure 3E). Further, a DCA was conducted to assess the decision benefit of the nomogram and the results suggested that it could accurately predict the 1-, 3-, and 5-year risks of patients with PAAD (Figure 3F).

### Functional enrichment in cuproptosis-related score

**FIGURE 4:**
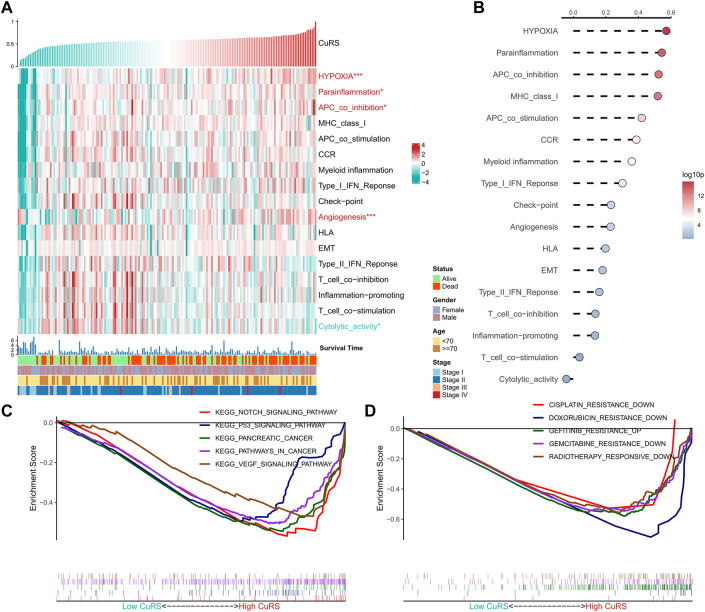
Functional analysis of the CRG-based risk model. **(A)** Heat map showing the correlation between CuRS, biological pathway activity, and clinical characteristics; **(B)** Correlation analysis for CuRS and biological pathways; **(C)** GSEA plot showing five KEGG pathways of interest in the high-CuRS group; **(D)** GSEA plot showing the responses of patients in the high-CuRS group towards chemotherapy and radiotherapy.

**FIGURE 5:**
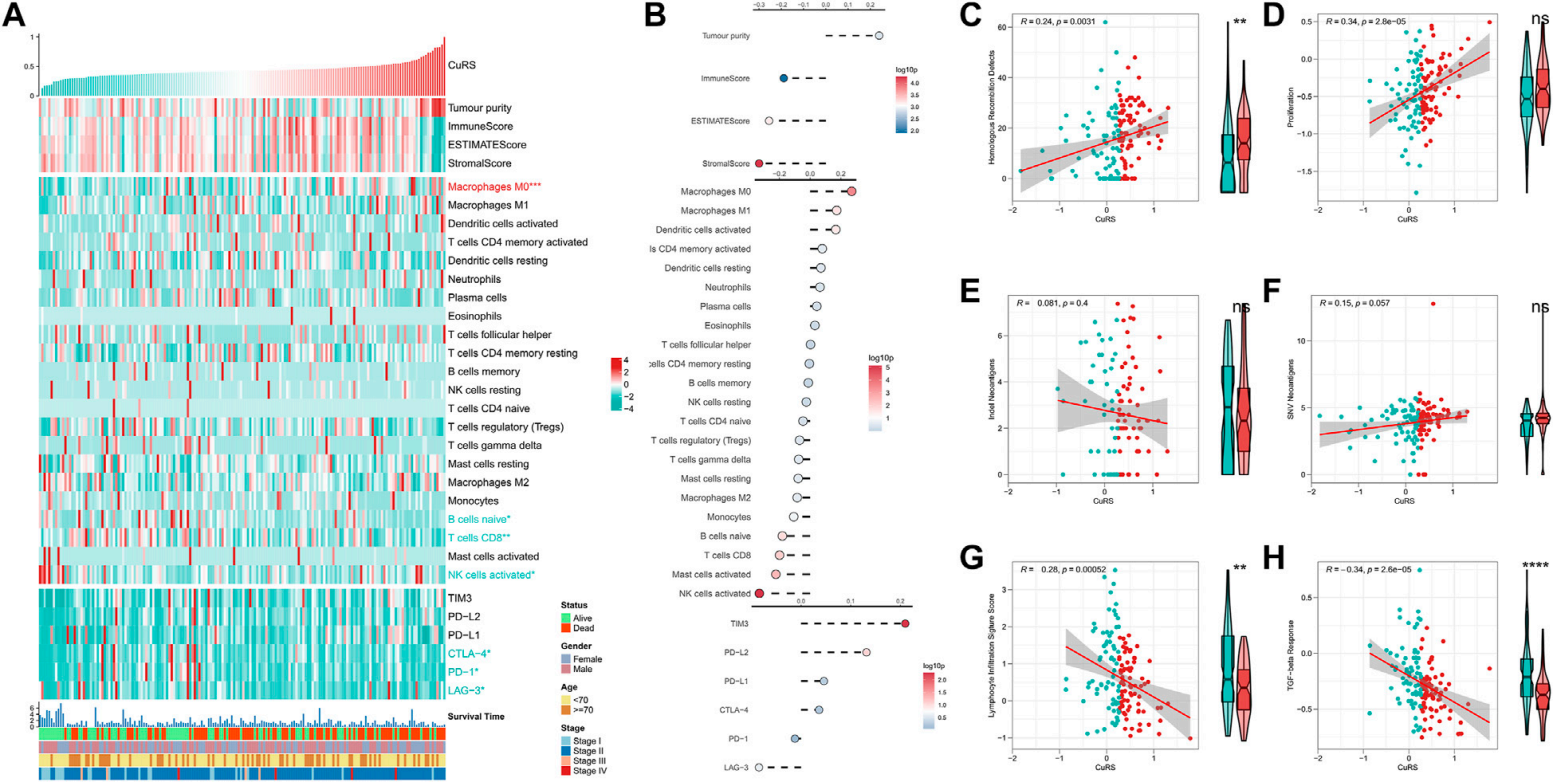
Immune landscape in the CuRS model. **(A)** Heat map showing the correlation between CuRS, estimate score, immune cell infiltration abundances, immune checkpoint expression, and clinical characteristics; **(B)** From top to bottom: correlation analysis for CuRS with estimate score, immune cell infiltration abundance, and immune checkpoint expression; Scatter and box plots showing the correlation of CuRS with **(C)** HRD score, **(D)** proliferation score, **(E)** intratumor heterogeneity score, **(F)** SNV neoantigens, **(G)** lymphocyte infiltration score, and **(H)** TGF-beta response.

The correlation between CuRS and multiple typical biological pathways was assessed. The heat map shows the relationship between CuRS, biological pathway activity, and clinical characteristics (Figure 4A). Results of the correlation analysis of CuRS with biological pathways are shown on the right side of the heat map (Figure 5B). Hypoxia, parainflammation, APC co-inhibition, and angiogenesis were significantly positively correlated with CuRS and significantly upregulated in the high-CuRS group, whereas cytolytic activity was negatively correlated with CuRS and significantly upregulated in the low-CuRS group. Further, GSEA showed that Notch, P53, and VEGF signaling pathways, along with PAAD-related pathways were significantly enriched in the high-CuRS group (Figure 4C). Finally, GSEA suggested that patients with a high CuRS were less resistant to cisplatin, doxorubicin, and gemcitabine but less sensitive to radiation and gefitinib treatment (Figure 4D).

### Immune landscape in the cuproptosis-related score model

The correlation between CuRS and the immune landscape was assessed. The heat map demonstrates the relationship between CuRS, estimate score, immune-infiltrating cell type abundances, and typical immune checkpoints (including PD-1, LAG-3, CTLA-4, PD-L1, TIM-3, and PD-L2), and clinical characteristics (Figure 5A). The corresponding results of correlation analysis are shown on the right side of the heat map (Figure 5B). Tumor purity was positively correlated with CuRS, whereas immune and estimate scores showed a negative correlation. However, no significant differences were observed between the two groups of patients. M0 macrophages were significantly positively correlated with CuRS and upregulated in the high-CuRS group. In contrast, B cells, CD8^+^ T cells, and NK cells were negatively correlated with CuRS and upregulated in the low-CuRS group. Additionally, TIM3 and PD-L2 were positively correlated with CuRS, whereas CTLA-4, PD-1, and LAG-3 were highly expressed in patients with a low CuRS. Cancers with homologous recombination (HR) defects suppress double-stranded DNA break repair. Therefore, such patients may show better sensitivity to DNA damaging agents, including platinum-based chemotherapy. Moreover, HRD scores were positively correlated with CuRS and markedly elevated in the high-CuRS group. In addition, tumor proliferation was also significantly positively correlated with CuRS (Figures 5C,D). However, indel and SNV neoantigens did not correlate significantly with CuRS, and lymphocyte infiltration scores along with the TGF-beta responses were significantly negatively correlated with CuRS and elevated in the low-CuRS group (Figures 5E–H). Moreover, CuRS was negatively correlated with the IPS; the low CuRS group showed high IPS (Figure 5I). Overall, CuRS could distinguish between “cold” and “hot” tumor subtypes, with lower CuRS suggesting stronger immunoreactivity (“hot” tumor), better patient survival, and more benefits from immunotherapy. In contrast, higher CuRS suggested more active proliferation (“cold” tumor), DNA damage, and benefits from chemotherapy.

### Correlation between cuproptosis-related score and somatic mutations

**FIGURE 6:**
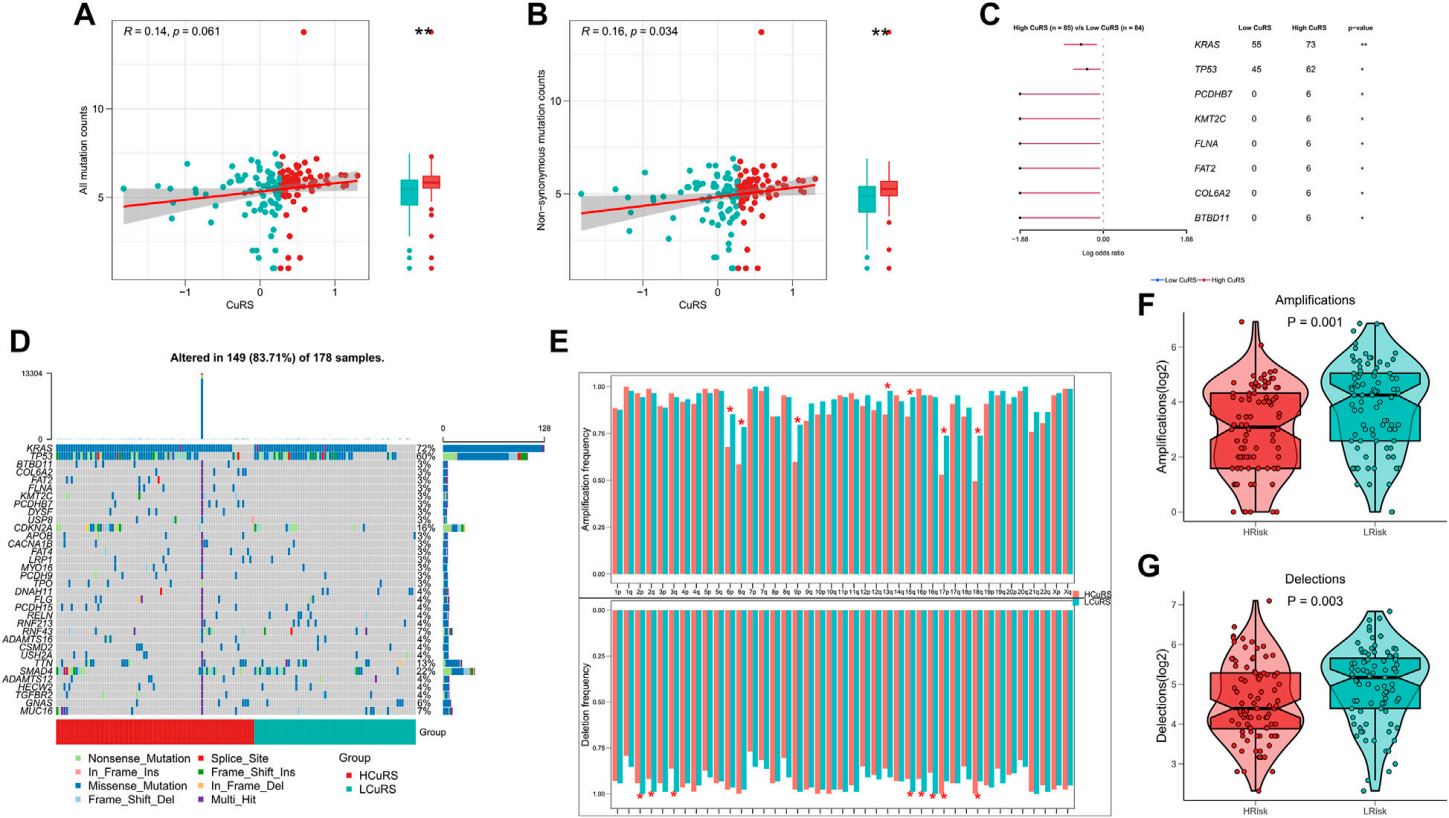
Genomic variation landscape for the CuRS model. **(A)** Correlation between CuRS and all types of mutation burdens; **(B)** Correlation between CuRS and non-synonymous mutation burden; **(C)** Forest plot showing significant differentially mutant genes (DMGs) between groups; **(D)** Oncoplot of DMGs between groups; **(E)** Bar chart showing CNV events on different chromosome arms in high- and low-CuRS groups (*p < 0.05); **(F)** Box plot showing the differences in chromosome amplification numbers between high- and low-CuRS groups; **(G)** Box plot showing the differences in chromosome deletion numbers between high- and low-CuRS groups.

TMB is associated with immunotherapeutic responses, whereby greater TMB may generate more potential neoantigens that can be recognized by the immune system. Antigens carrying mutant peptides, after recognition, can activate the immune system and enhance anti-tumor immunity (Matsushita et al., 2012; Rizvi et al., 2015; Chan et al., 2019). Based on the clinical value of TMB, we examined the correlation between TMB and CuRS. The results suggested that all types of mutational burdens and non-synonymous mutational burdens were elevated in the high-CuRS groups. However, only non-synonymous mutations showed a positive correlation with CuRS (Figures 6A,B). Additionally, the differences in mutation frequencies among high-frequency mutant genes relative to the low-CuRS group were compared. The Forest plot suggested that the mutation frequencies of KRAS, TP53, PCDHB7, KMT2C, FLNA, FAT2, COL6A2, and BTBD11 were significantly higher in the high-CuRS group as compared to those in the low-CuRS group (Figure 6C). Figure 6D illustrates the mutation landscape in both groups. CNV caused chromosomal variations differently. Finally, we assessed the correlation between CuRS and CNV events. Overall, more CNV events were observed on the chromosomal arms in the low-CuRS group (Figure 6E). Box plot showed significantly more deletion and amplification events in the low-CuRS group (Figures 6F,G).

### Role of cuproptosis-related score in clinical decision-making

**FIGURE 7:**
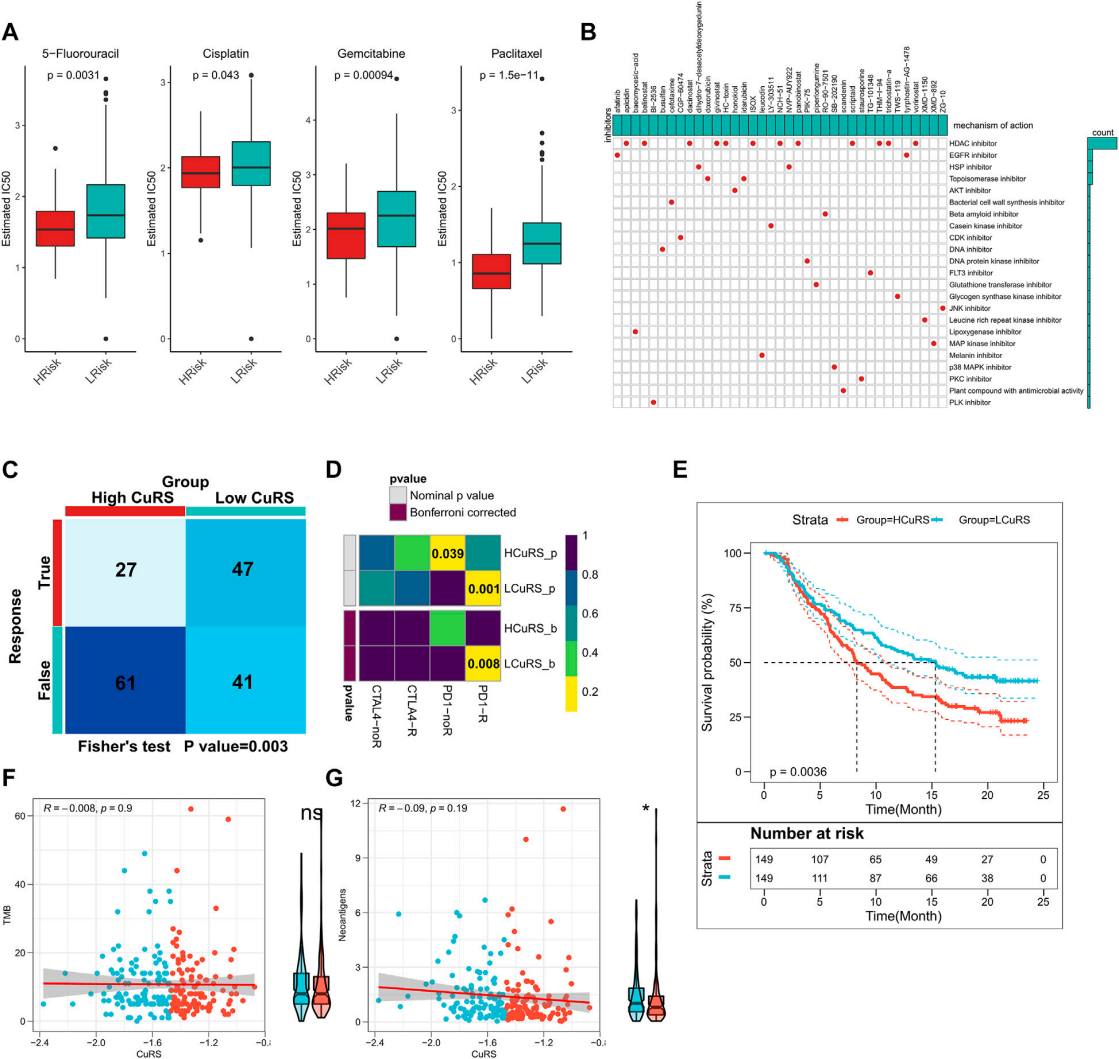
Role of CuRS in clinical decision-making. **(A)** Box plot showing the predicted IC_50_ values for four commonly used drugs between the high- and low-CuRS groups; **(B)** Oncoplot showing the identified small molecule compounds, where the horizontal axis represents the name of the small molecule inhibitor and the vertical axis represents the biological pathway targeted by the corresponding small molecule inhibitors; **(C)** TIDE algorithm to predict the responses of patients in the high- and low-CuRS groups to immunotherapy; **(D)** Subclass mapping to predict the sensitivities of patients in the high- and low-CuRS groups to PD1 and CTLA4 treatments; **(E)** KM survival curves for the high- and low-CuRS groups in the IMvigor210 cohort; **(F)** Correlation between CuRS and TMB in the IMvigor210 cohort; **(G)** Correlation between CuRS and neoantigens in the IMvigor210 cohort.

The above results suggested that patients with high- and low-CuRSs were more sensitive to chemotherapy and immunotherapy, respectively. Differences in patient sensitivity to commonly used chemotherapeutic agents were assessed and those in the low-CuRS group in the TCGA cohort were found to be more sensitive to 5-FU, cisplatin, gemcitabine, and paclitaxel (Figure 7A). Similar results were observed in the validation cohort (Supplementary Figures S3A,B). A total of 37 small molecule drugs effective in patients with a high CuRS were subsequently identified to target 23 biological pathways (Figure 7B). Subsequently, patient responses to immunotherapy were assessed using the TIDE algorithm. Patients with lower CuRSs showed higher responses in both TCGA (*p* = 0.003, Figure 7C) and external validation cohorts (*p* < 0.05, Supplementary Figures S2C,D). Additionally, subclass mapping results suggested that patients with lower CuRSs showed increased sensitivity to anti-PD1 therapy in both TCGA and external validation cohorts (FDR <0.01) (Figure 7D; Supplementary Figures S3E,F). Moreover, the CuRS model was constructed for the immunotherapy cohort, IMvigor210, which revealed significantly worse survival among patients in the high-CuRS group (*p* = 0.0036, Figure 7E). Subsequently, the relationship of TMB and neoantigens with CuRS in the immunotherapy cohort was assessed. The results suggested that neoantigen expression was significantly higher in the low-CuRS group. However, TMB did not exhibit a significant correlation with CuRS (Figures 7F,G). Overall, these findings suggested that the CuRS model was a viable tool to guide clinical treatment decisions for patients with PAAD.

## Discussion

A novel CuRS model incorporating seven genes was constructed in this study. CuRS was an independent prognostic factor, whereby a higher CuRS predicted a worse prognosis. Patients in the high-CuRS group showed higher hypoxic and angiogenic activities, lower levels of immune cell infiltration, lower immunogenicity, lower immune checkpoint activity, higher tumor purity, and higher genomic alteration status as compared to those in the low-CuRS group. Additionally, patients with high CuRSs exhibited increased sensitivity to conventional chemotherapy but poorer responses to immunotherapy.

Cell death is significantly associated with cancer progression, metastasis, and treatment responses. Inhibition of cell death enhances tumor metastasis and resistance to chemotherapy in malignant cells (Su et al., 2015; Strasser and Vaux, 2020). As most tumors are innately resistant to apoptosis, the induction of non-apoptotic cell death has emerged as a new strategy in cancer treatment (Tang et al., 2020). PAAD is a malignant tumor and multiple mechanisms of resistance to apoptosis result in low sensitivity to conventional chemotherapy and radiotherapy regimens in these patients (Hamacher et al., 2008). Other cell death-related mechanisms, including ferroptosis and pyroptosis, can be targeted for PAAD treatment (Chen et al., 2021; Ye et al., 2021; Yu et al., 2022). Cuproptosis is a novel form of cell death and plays a role in tumors that are innately resistant to apoptosis (Kahlson and Dixon, 2022; Tsvetkov et al., 2022). This is the first study to focus on cuproptosis as a cell death mechanism in PAAD. Our findings suggested that DLAT, GSS, NDUFB2M, and TIMMDC1 were significant prognostic factors. Additionally, the NRG-based risk model exhibited excellent predictive performance in both the training and the external validation cohorts.

This study confirmed significant differences in biological pathways between the two groups. Patients in the high-CuRS group showed significantly higher angiogenic and hypoxic activities. Previous studies report that active angiogenesis is essential for tumor growth and metastasis, thus resulting in immune function suppression; therefore, angiogenesis inhibition is a promising therapeutic option for suppressing tumor growth (Sharma et al., 2001; Motz and Coukos, 2011; Welti et al., 2013). Hypoxia can suppress TME and promote PAAD progression (Liu et al., 2019; Gupta et al., 2021; Tao et al., 2021). Cancer-related pathways such as P53 and VEGF were enriched in the high-CuRS group. These findings suggested that a high CuRS indicated a higher degree of PAAD malignancy. The elevated cell killing activity in the low-CuRS group suggested high anti-tumor immune responses (Sivori et al., 2021; You et al., 2021). Overall, patients in the high-CuRS group experienced tumor growth and immunosuppression resulting in significantly poorer survival, whereas those in the low-CuRS group exhibited stronger anti-tumor immunity.

The synergistic effects of TME and immunoreactivity are significantly associated with cancer treatment and patients’ prognoses (Bruni et al., 2020; Riera-Domingo et al., 2020). In this study, we assessed the differences in TME and immunoreactivity between the two groups. Patients in the low-CuRS group exhibited higher immune scores and immune checkpoint activity, indicating stronger immune functions. Additionally, high CuRSs were associated with higher M0 macrophage activity, whereas low CuRSs were associated with higher CD8 T, B, and NK cell activities. These findings suggested that high CuRSs may contribute to an immune-silenced tumor phenotype, whereas low CuRSs lead to an immune-activated phenotype with active anti-tumor immune responses (Hamanishi et al., 2007; Thorsson et al., 2018; Bald et al., 2020), consistent with a better survival status among patients in the low-CuRS group. Moreover, patients in the low-CuRS group may develop “hot” tumors that are sensitive to immunotherapy. In addition, high CuRSs represent a high HRD score, leading to impaired double-strand break repairs, a common driver of tumorigenesis (Nguyen et al., 2020). HRD score is highly correlated with the clinical progression of PAAD (Wagener-Ryczek et al., 2021). Further, high CuRS was associated with an increased proliferation score, suggesting a high tumor cell malignancy. Although neoantigens did not exhibit significant differences between the two groups, lymphocyte infiltration scores and TGF-beta responses were significantly associated with low CuRS. Similarly, more tumor-infiltrating lymphocytes lead to stronger anti-tumor immunoreactivity and responses to immunotherapy (Waldman et al., 2020; Paijens et al., 2021). TGF-beta plays a dual role in PAAD by mediating tumor-stromal crosstalk and regulating TME (Qian et al., 2020). These findings suggested that low CuRS could predict immune activation in TME and the development of immunotherapy-sensitive “hot” tumors.

TMB is a biomarker of patients’ responses to immunotherapy, and higher TMB suggests better immunotherapeutic outcomes (Hellmann et al., 2018a; Hellmann et al., 2018b). In this study, patients in the high-CuRS group exhibited high TMB but low immunoreactivity, suggesting that high TMB was not necessarily predictive of high immunogenicity. TMB is reportedly inefficient in predicting the potential benefits of immunotherapy among patients with ADD (Eso and Seno, 2020; Lawlor et al., 2021). Our results suggested that CuRS and TMB represent different aspects of tumor immunobiology in PAAD and the former could better identify “hot” tumors with an immune-activated phenotype.

Immunotherapy is a novel therapeutic strategy for treating multiple cancers including PAAD. Identifying patients who can benefit from immunotherapy remains a great challenge. PD-1 expression, microsatellite instability, and mutation burden are inefficient in predicting the potential benefits of immunotherapy (Lawlor et al., 2021). In this study, the accuracy of CuRS in predicting patients’ responses to immunotherapy was assessed by multiple methods. TIDE and subclass mapping analyses suggested that patients with higher CURSs were more sensitive to anti-PD1 therapy, which was confirmed in an external validation cohort. Evaluation of patients who received anti-PD1 immunotherapy in the IMvigor210 cohort showed significantly better survival among those with low CuRSs. Additionally, patients in the low-CuRS group showed significantly higher neoantigens. Drug sensitivity analysis suggested that CuRS may facilitate chemotherapy. Some drugs commonly used in PAAD treatment, such as 5-FU, gemcitabine, and paclitaxel, were more effective among patients in the high-CuRS group. These results suggested that cuproptosis could affect the efficacy of chemotherapeutic agents. Furthermore, GSEA suggested that patients in the high-CuRS group showed reduced responses to radiotherapy. Therefore, cuproptosis-based strategic optimization of chemotherapy, radiotherapy, and immunotherapy proposed in this study may be effective in treating PAAD. Previous studies have focused on the association between pyroptosis and ferroptosis in the treatment of PAAD. In comparison, cuproptosis shows better efficacy in decision-making for treatment regimens, especially chemotherapy (Yu et al., 2022).

However, this study has some limitations. First, the lack of data resulted in only inter-patient heterogeneity being accounted for, and not intratumoral heterogeneity. Additionally, although we have used some algorithms to assess the accuracy of this risk model in predicting patient sensitivity to chemotherapy and immunotherapy, further validation in prospective cohort trials and clinical data is warranted in the future. Moreover, changes in the immune microenvironment are dynamic; however, we have only discussed the heterogeneity of the immune microenvironment. Additional time series experiments can better explain the dynamic interactions between cuproptosis and the immune microenvironment. Finally, *in vivo* and *in vitro* experiments are needed to assess the specific biological functions of cuproptosis in PAAD.

In conclusion, a novel CuRS model was developed in this study to predict the OS of patients with PAAD, which was validated in the training and external validation cohorts. Low CuRSs suggest active anti-tumor immunity and stronger immune activation (“hot” tumors). Additionally, this model could predict patient sensitivities to chemotherapy and immunotherapy. Overall, the findings of this study contribute to further understanding of cuproptosis and the development of precise PAAD treatment.

## Data Availability

The original contributions presented in the study are included in the article/Supplementary Material, further inquiries can be directed to the corresponding author.

## Supplementary material

Supplementary Figure S1 External validation of the CuRS model. KM survival curves for patients in the **(A)** ICGC and **(B)** E-MTAB cohorts; Survival status of patients and expression of model genes in the **(C)** ICGC and **(D)** E-MTAB cohorts; **(E)** 1-, 3-, 5-, and 8-year ROC curves for CuRS in the **(E)** ICGC and **(F)** E-MTAB cohorts.

Supplementary Figure S2 Subgroup Cox analysis for the CuRS model. Subgroup Cox regression analysis of CuRS in **(A)** TCGA, **(B)** ICGC, and **(C)** E-MTAB cohorts.

Supplementary Figure S3 External validation of CuRS model-related treatment decisions. Predicted IC_50_ values for the four commonly used drugs in the high- and low-CuRS groups in the **(A)** ICGC and **(B)** E-MTAB cohorts; differences in immune responses predicted by the TIDE algorithm between the high- and low-CuRS groups in the **(C)** ICGC and **(D)** E-MTAB cohorts; Immune response differences predicted by subclass mapping between the high- and low-CuRS groups in the **(C)** ICGC and **(D)** E-MTAB cohorts.
