# Supplementary figures and images for: Cuproptosis scoring model predicts overall survival and assists in immunotherapeutic decision making in pancreatic carcinoma
Source: Front Genet. 2022 Aug 31;13:938488. doi: 10.3389/fgene.2022.938488 (PMC9472214; doi:10.3389/fgene.2022.938488)

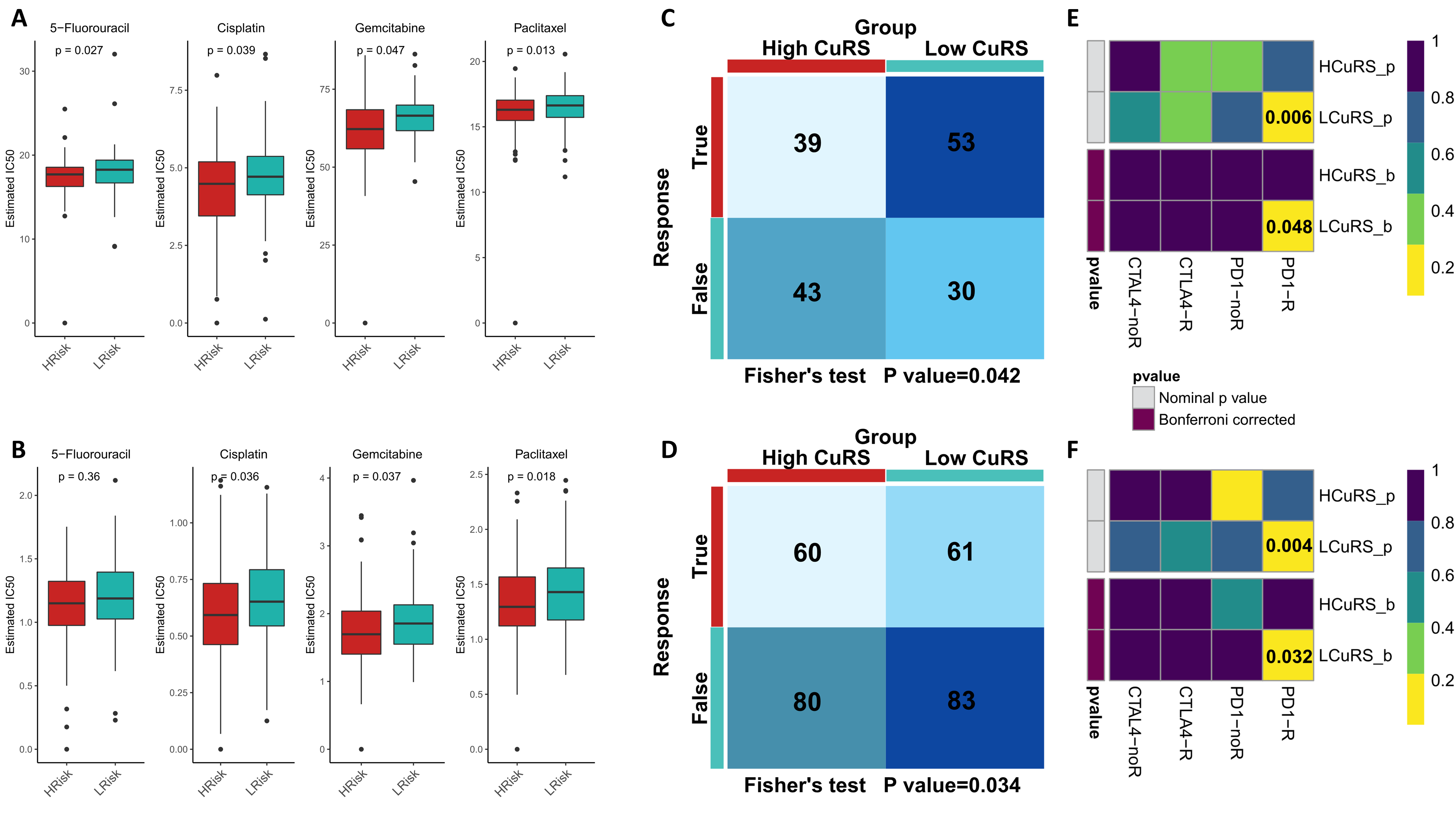

Supplement: Supplementary file 3 [file Image3.tif]

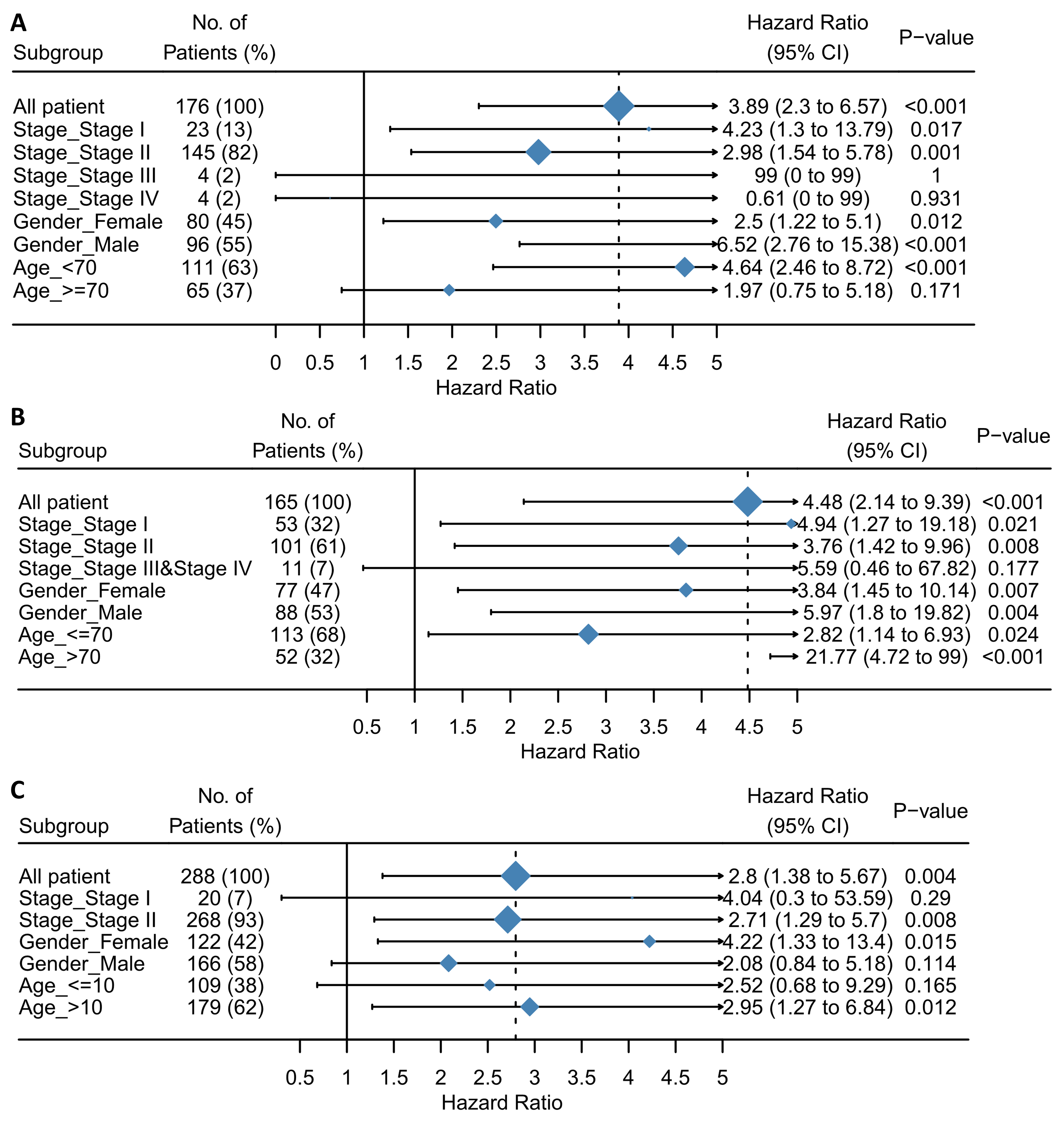

Supplement: Supplementary file 4 [file Image2.tif]

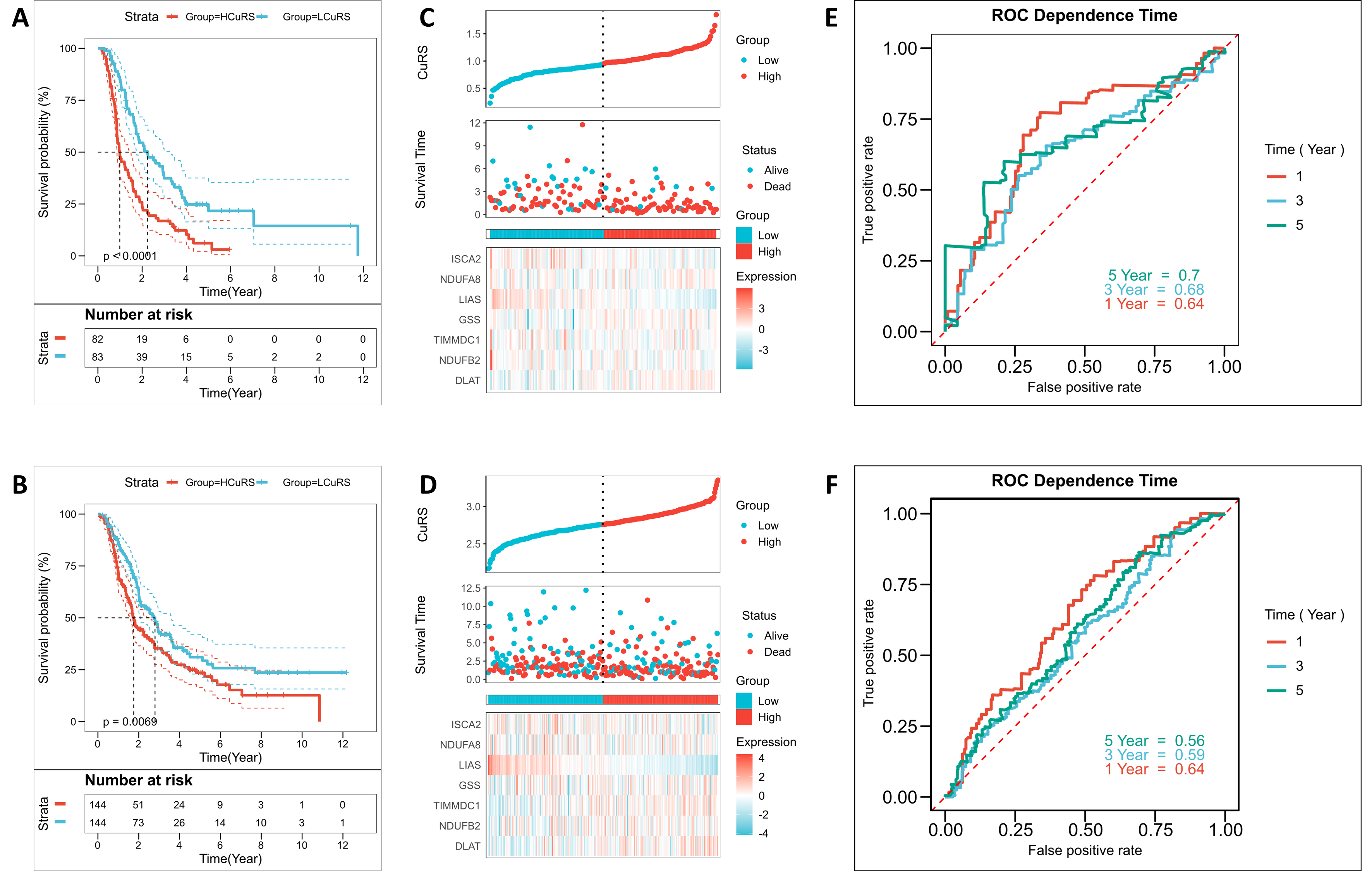

Supplement: Supplementary file 5 [file Image1.tif]
